# Community knowledge and practices regarding malaria and long-lasting insecticidal nets during malaria elimination programme in an endemic area in Iran

**DOI:** 10.1186/1475-2875-13-511

**Published:** 2014-12-24

**Authors:** Mussa Soleimani-Ahmadi, Hassan Vatandoost, Mehdi Zare, Ali Alizadeh, Mehrdad Salehi

**Affiliations:** Infectious and Tropical Diseases Research Center, Hormozgan University of Medical Sciences, Bandar Abbas, Iran; Department of Medical Entomology and Vector Control, School of Public Health, Hormozgan University of Medical Sciences, P.O. Box: 79145–3838, Bandar Abbas, Iran; Department of Medical Entomology and Vector Control, School of Public Health and National Institute of Health Research, Tehran University of Medical Sciences, Tehran, Iran; Department of Occupational Health Engineering, School of Public Health, Hormozgan University of Medical Sciences, Bandar Abbas, Iran; Social Determinants in Health Promotion Research Center, Hormozgan University of Medical Sciences, Bandar Abbas, Iran; Rudan Health Center, Hormozgan University of Medical Sciences, Rudan, Iran

**Keywords:** Malaria, Knowledge, Practice, Long-lasting insecticidal nets, Rudan, Iran

## Abstract

**Background:**

Since malaria is one of the foremost public health problems in Iran, a malaria elimination phase has been initiated and application of long-lasting insecticidal nets (LLINs) is an important strategy for control. Success and effectiveness of this community based strategy largely dependent on proper use of LLINs. In this context, to determine the community’s knowledge and practices about malaria and LLINs, a study was conducted in Rudan County, one of the important malaria endemic areas in southeast of Iran.

**Methods:**

In this cross-sectional study, 400 households in four villages were selected by cluster randomly sampling method. Community knowledge and practices about malaria and LLINs including symptoms and transmission of malaria and washing, drying and using of bed nets were investigated using pre-tested structured questionnaires. The data were analysed using SPSS.16 software.

**Results:**

In this study nearly 89% of the respondents knew at least one symptom of malaria and 86.8% considered malaria as an important disease. The majority of respondents (77.8%) believed that malaria transmits through mosquito bite and 72.5% mentioned stagnated water as a potential mosquito breeding place. About 46% of respondents mentioned the community health worker as the main source of their information about malaria. Approximately 44.8% of studied population washed the LLINs once in six months and 92% of them mentioned that they dry the bed nets in direct sunlight. While 94% of households reported they received one or more LLINs by government and 60.8% of respondents mentioned that LLINs were the main protective measure against malaria, only 18.5% of households slept under bed nets the night before the survey, this use rate is lower than the targeted coverage (80%) which is recommended by World Health Organization.

**Conclusion:**

Although, majority of studied population were aware of the symptoms and cause of malaria, a majority had misconceptions about LLINs. Therefore, appropriate educational intervention by trained health workers should be developed for a behaviour change and motivating people to use LLINs which would improve malaria elimination programme.

**Electronic supplementary material:**

The online version of this article (doi:10.1186/1475-2875-13-511) contains supplementary material, which is available to authorized users.

## Background

Malaria is considered to be the most prevalent vector-borne disease worldwide and is currently endemic in 97 countries [1]. The disease is endemic in the southeast of Iran, with two seasonal peaks mainly in spring and autumn. The current strategic approaches to malaria control emphasize prevention through the use of long-lasting insecticidal nets (LLINs), as recommended by the World Health Organization (WHO) [2].

Iran has initiated measures to improve malaria elimination through using larvicides, indoor residual spraying (IRS), LLINs, and early case detection and treatment of malaria. In this regard, Iran is aiming to eliminate *Plasmodium falciparum* by 2015 and to become malaria-free by 2025 [2, 3]. Since 2008, Iran is receiving a Global Fund grant to develop vector control activities, such as distributing insecticide-treated nets (ITNs) and targeted use of IRS [2, 4]. Adequate knowledge on malaria transmission is essential for effective use of preventive measures. People perceive the direct benefits of bed nets through an observable reduction of mosquito nuisance and malaria episodes, which may motivate them to use bed nets more effectively [5]. Besides assuring access to LLINs, acceptability and compliance with net use are the other critical issues in the success of any ITNs programme [6].

The results of studies in various parts of the world revealed that use of insecticide-treated nets is an effective tool against anopheline mosquitoes and reduces morbidity and mortality due to malaria [7, 8]. Treated bed nets have an influential impact on mosquito density and sporozoite rates. However, effectiveness of bed net use is dependent on attitudes and socio- cultural context of population [9]. A high coverage of long- lasting insecticidal nets followed by educational intervention, results in a decrease in malaria morbidity and reduces transmission, as showed in a study from rural area in southeast of Iran [10]. A recent study on malaria knowledge in Iran indicated that populations’ educational level is positively associated with knowledge of malaria transmission, aetiology and clinical symptoms of the disease [11].

Rudan is a malarious area with local transmission in the southeast of Iran. In this county, 63 cases of malaria were reported during 2009–2013. The parasite species composition was 91% *Plasmodium vivax* and 9% *Plasmodium falciparum* (Hormozgan Health Centre, unpublished data, 2014). Previous studies have reported four malaria vectors in this area including: *Anopheles culicifacies s.l.*, *Anopheles dthali*, *Anopheles stephensi* and *Anopheles superpictus* where, *An. stephensi* is primary vector and other species play role as secondary vectors [12].

Indoor residual spraying and distribution of long-lasting insecticide-treated nets have been the main vector control measures implemented as part of an integrated approach to malaria elimination in this county [12]. In addition to achieve sufficient LLINs coverage, a challenge is identifying and addressing the behavioural factors that influence LLINs use. Understanding the community knowledge about malaria and LLINs would help in designing sustainable malaria control programmes that will lead to behavioural change and adoption of new ideas [13]. The participation of the community is one of the major tools of malaria elimination programmes and improved community knowledge of malaria and its transmission can promote preventive and personal protective practices amongst the target populations [14]. Parallel to implementation of malaria elimination in Iran, this study was conducted to determine the community knowledge and practice regarding malaria and its preventive measures, with an emphasis on the use of LLINs in Rudan County, southeast of Iran.

## Methods

### Study area

The study was carried out in Rudan county of Hormozgan province, one of the malaria endemic areas in southeastern Iran. The county is located between 27°05′- 27°59′ N latitudes and 56°50′ - 57° 29′ E longitudes with an estimated population of 118,547 individuals in 2011. The annual mean relative humidity is 54% and the average of annual rainfall is about 250 mm. The average daily temperature ranges from 9°C to 45°C. In the study area nearly all of houses are made of cement blocks and have electricity and water supply. The main economic activities in the area are agricultural with palm and citrus plantation and livestock herding. Malaria transmission in the area is endemic which occurs year-round with peaks after the two annual rainy seasons (April-June and October -December) [3].

### Study design and data collection

The study was a community-based cross-sectional survey conducted between May and July, 2013 in Rudan County, where LLINs had been previously distributed for malaria elimination.

Assuming expected knowledge about malaria and LLINs to be 50% and a desired precision of 5%, the sample size calculated by the formula to be 400. Study villages were selected by a two-stage randomized cluster sampling procedure. In the first stage, four villages with similar topographical and epidemiological situations were selected randomly. In the second stage, 100 households were randomly selected for each village. As the father of family is mostly out of house to work, mothers of randomly selected households were interviewed using a pre-tested, structured questionnaire (see Additional file 1). In case they were absent, the daughter of household or another adult member was interviewed instead. The questionnaires were administered by trained field interviewers and supervised by the principal investigator. The questions included respondents’ demographic characteristics, knowledge (five items) and practices (10 items) about malaria and LLINs including malaria symptoms and transmission and washing, drying, and using of bed nets.

Interviewers verified the bed net usage during last night by checking whether the net was hanging over sleeping place or folded during the home visit in the morning hours. The answers about dwelling houses construction materials, windows, and water containers were confirmed during the investigators visit of each household. In addition, socio-economic factors which could predispose communities to malaria transmissions were investigated.

### Statistical analysis

The data were analysed using SPSS version 16. Descriptive statistics were used to measure percentages, averages, and relative frequencies of the variables. Cross tabulations of variables were done and Chi-squared test (*χ*^2^) was used to determine the statistical significance of differences of relative frequencies. The results were considered significant at 5% levels of significance (p < 0.05). The knowledge and practice regarding malaria and LLINs were compared with the educational status of the respondents. Correlation coefficients were calculated for the knowledge regarding malaria, LLINs and the reported practices.

### Ethical consideration

Households of study villages were informed about the objectives and procedures of the investigation. The respondents were informed that their participation was purely voluntary and they were free to withdraw from the study at any time. Study identification numbers were used instead of participant names and collected data were kept confidential.

## Results

### Characteristics of the study population

A total of 400 households were interviewed. The ages of respondents ranged from 17 to 75 years with an average of 37.07 years. The mean family size was 4.9 people ranging from 1 to 11 people. About one third of the women had completed primary school education (32.3%) and 37.3% had not attended any formal education. Most of the respondents (92.7%) were unemployed and engaged in housework; others were self-employed, farmer/stockbreeder, students, and in other services. Socio-demographic Characteristics of the study population is shown in Table 1. The majority of households had a home constructed of cement blocks (96.7%) and 14.5% of houses had screens over the window openings (Table 2). Most of the study population had access to clean piped water (96.5%) and electricity (98.0%) in their houses. About half of houses (49.2%) were built within 20 metres of domestic animal shelters (Table 2).

**Table 1:** **Socio-demographic characteristics of the study population in Rudan County, southeast of Iran**

Characteristics	Number	Percent
**Ages groups (years)**		
15-24	66	16.5
25-34	112	28
35-44	116	39
45+	106	26.5
**Education**		
Illiterate	149	37.3
Primary	129	32.3
Secondary	52	13.0
High school	56	14.0
University	14	3.4
**Family size**		
1-2	52	13.0
3-4	170	42.5
5-6	124	31.0
7+	54	13.5
**Occupation**		
Housewife	371	92.7
Employed	10	2.5
Self-employed	2	0.5
Farmer/Stockbreeder	9	2.3
Student	8	2.0

**Table 2:** **Characteristics of residence houses in the study area in Rudan County, southeast of Iran**

Characteristics	Yes		No	
	Number	Percent	Number	Percent
**Type of house**				
Cement block house	387	96.7	13	3.3
Shed	11	2.8	389	97.2
Tent	2	0.5	398	99.5
**Situation of house**				
Window screens	58	14.5	342	85.5
Water supply	386	96.5	14	3.5
Water saving container	102	25.5	298	74.5
Electricity	392	98.0	8	2.0
Air conditioner	363	91.0	37	9.0
Animal shelter close to house	198	49.2	202	50.8

### Malaria knowledge and practices

The majority of study population (86.8%) had knowledge about malaria as a disease and 77.8% of them believed that malaria is transmitted through the bite of mosquitoes and this was significantly associated with their educational level (*X*^2^ = 3.84, df = 4, P = 0.038) (Table 3). Even though a majority of respondents were aware of the cause of malaria, 8% didn’t know the actual cause of malaria and 14.2% had misconceptions, such as drinking dirty water, eating contaminated food and inhaling polluted air as the cause of malaria transmission (Table 3).

**Table 3:** **Knowledge and practices regarding malaria in the study population in Rudan County, southeast of Iran**

Parameters	Number	Percent
**Malaria transmission**		
Mosquito bites	311	77.8
Drinking dirty water	38	9.5
Eating contaminated food	11	2.7
Inhaling polluted air	8	2
Don’t know	32	8
**Malaria symptoms**		
Fever	67	16.7
Chill	5	1.3
Fever and chill	275	68.7
Bone pain	36	9
Abdominal discomfort	9	2.3
Don’t know	8	2
**Malaria preventive measures**		
Use of long- lasting insecticidal nets	243	60.8
Use of indoor residual spraying	60	15
Chemoprophylaxis	18	4.5
Use of door/window screens	58	14.5
Others	13	3.2
Noting	8	2
**Mosquito breeding places**		
Stagnant water	290	72.5
Rubbish	49	12.3
Don’t know	51	12.7
Others	10	2.5
**History of malaria infection in family members**	40	10
**Interesting in participation in malaria control programmes**	204	51

Fever and chills were the two most frequently mentioned signs and symptoms of malaria, although participants also identified bone pain and abdominal discomfort as other symptoms of the disease (Table 3).

Nearly 10% of participants reported having cases of malaria infection in their family within the past 3 years (Table 3). The households who had previously been infected with malaria in their family showed better knowledge of malaria symptoms than those with no history of malaria infection (*X*^2^ = 8.32, df = 6, P = 0.02). Most of the respondents (72.5%) considered stagnated water as breeding place of mosquitoes.

**Figure 1:**
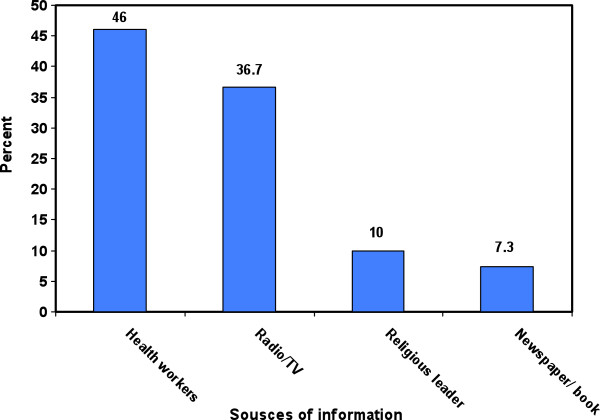
**Sources of information about malaria in the study population in Rudan County, southeast of Iran.**

Generally, community knowledge regarding malaria prevention was high (98%) and most of households (60.8%) mentioned the use of LLINs as a preventive measure against malaria. Other mentioned measures against malaria included indoor residual spraying (15%), screen on doors/windows (14.5%) and chemoprophylaxis (4.5%) (Table 3).The community health workers were the most prominent source of malaria information (46%) and media, i.e., television and radio were the second most mentioned sources of information (36.7%). In addition, some of participants received malaria information from religious leaders in community meetings. Newspapers and books were the least often heard messages (Figure 1).

This study results also showed that about half of the respondents (51%) were interested in participating in malaria control programme as a volunteer (Table 3). A significant relationship was found between the educational levels of respondents and their interest in participating in malaria control programmes as a volunteer (X ^2^ = 12.18, df = 6, P = 0.014).

### Knowledge and practice regarding LLINs

Out of 400 studied households 376 (94%) reported they received one or more LLINs by government. The majority of households (76.8%) reported that they use LLINs. However, regular use of LLINs was 18.5% as it was checked visually in the morning hours by interviewers (Table 4). Analysis of factors influencing regular use of bed nets showed that bed net use rate was positively associated with educational level of respondents (*X*^2^ = 6.82, df = 2, P = 0.001).

**Table 4:** **Knowledge and practices regarding long-lasting insecticidal nets in the study population in Rudan County, southeast of Iran**

Parameters	Number	Percent
**Frequency of bed nets use**		
Regular use (Bed net used every night)	74	18.5
Irregular use (Missed using bed net in some time)	233	58.3
Not using	93	23.2
**Family members who use bed nets**		
All family members	330	82.5
Children	32	8
Father and mother	38	9.5
**Bed nets use during night**		
All the time at night	173	43.5
Only when sleeping	227	56.5
**Reason for use of bet nets**		
Prevention of mosquito nuisance	336	84
Prevention of other insects nuisance	34	8.5
Prevention of scorpion stings	22	5.5
Others	8	2
**Washing frequency of bed nets**		
Once in a month	53	13.2
Once in a six months	179	44.8
Once in a year	148	37
Not washing	20	5
**Drying the bed nets**		
Dry in the sunlight	368	92
Dry in the shade	32	8

Most of the studied population (82.5%) reported that all of family members sleep under bed nets and 17.5% of respondents mentioned that only parents (9.5%) or children (8%) use bed nets. There was no association between using bed nets and reporting cases of a malaria infection in the family in the past year (*X*2 = 3.26, P = O.812).

The results also showed that 43.5% of households use the bed nets all the time at night and 56.5% of them over the sleeping time (Table 3). Protection against mosquito bites and malaria transmission was reported to be the main reason for using LLINs (84%). The other reasons were having an undisturbed night sleep with protection from other insects’ nuisance (8.5%) and protection from scorpion stings (5.5%).

In this study, approximately 87% of households reported they did not receive instructions on washing and drying the bed nets at the time of LLINs distribution. The results also showed that 44.8% of studied population wash the LLINs once in six months. Moreover, the majority of respondents (92%) mentioned that they dry the washed LLINs in direct sunlight because it is a usual practice for drying wet cloths. Details of net washing and drying practices are shown in Table 4.

## Discussion

This study was conducted to provide baseline information on knowledge and practices regarding malaria and LLINs, which can be used in the development of a plan for community participation towards the prevention and control of malaria. This study indicated that the knowledge of studied population about malaria transmission and symptoms is high. This finding is supported by other studies in the southeast of Iran [11]. Similarly, in studies conducted in Ghana, Malaysia, Tanzania, Guinea-Bissau, Mali and Uganda high awareness of people about malaria was reported [13–19].

According to the results, the most important sources of information were health centres. This finding illustrates that health workers are frequently in contact with villagers. It has been previously reported that access to health centres and communication facilities plays an important role in prevention and control of malaria [19].

This study also showed a positive relationship between knowledge of the respondents about malaria symptoms and the past history of malaria infection in the family. It is a common observation in endemic area where people suffer frequently from malaria infection [20–22].

In this study, majority of the people knew that mosquito bite can cause malaria. High level of knowledge about malaria transmission route in the studied population may be due to long-term exposure to malaria over years. In contrast to these findings, in other communities only those with a higher level of education knew the vector and symptoms of malaria [14, 23].

An important finding of this study was that majority of the households knew stagnated water as breeding place of mosquitoes. This finding is in agreement with a previous study conducted in Bashagard county, adjacent to the study area, which revealed high knowledge of people about mosquito breeding places [10]. Similar findings have been reported from malaria endemic countries such as Tanzania, Nepal and India [20, 24, 25]. High awareness of mosquitoes breeding places could influence several factors involved in the vector control, such as the selection of residential areas and use of preventive measures aiming to reduce mosquito population density.

According to the results, although 77.8% of the studied population mentioned mosquitoes as the vector of malaria and 60.8% reported LLINs as the preventive measure against malaria transmission, LLINs were used by only 18.5% when interviewers checked bed nets use visually in the morning hours. A reason for this may be low perceived susceptibility of households about malaria infection; in other words, they don’t see themselves exposed to malaria infection. Another reason can be their low perceived severity of malaria infection. A comparable observation has been made in rural community of western Kenya [26]. Our results are also similar to findings of Tsuyuoka *et al.* in Zimbabwe, where 6- 24% of children under five years and 2.8 - 9.7% pregnant women used [27].

An LLIN use rate of 18.5%, as found in this study, is thus considerably lower than 80% which is the targeted coverage of the Roll Back Malaria [28]. Moreover, this is much lower than LLINs use rate in other malaria endemic countries, such as India (79.2%), Sierra Leone (67.2%), and Sri Lanka (90%) [6, 29, 30]. Moreover, 56.5% of households reported they use bed nets only during sleeping time. Considering that malaria transmission occurs during 10 months in the study area and some of malaria vectors prefer to bite mainly outdoors in the earlier times of night [4, 12, 31], peoples who spend earlier times of night outside may be bitten more frequently and have more chance to get malaria. There were several reports confirming that regular use of insecticide-treated nets increase when individuals receive information about bed nets [8, 13]. In a recent study conducted in a malarious area of Iran, regular use of LLINs increased from 58.3% to 92.5% following educational intervention [10]. Therefore, effective educational programmes may increase use of LLINs in the studied population.

According to the results about half of households washed their LLINs once in six months which is according to the manufacturer’s maintenance instructions [8]. They had not received manufacturer’s washing instructions and the main reason for washing frequency was that they became dirty. Similar findings have been reported from malaria endemic areas of the Sri Lanka [32]. Hence, required washing frequency of bed nets should be instructed to the studied population during an educational intervention.

Previous study in the southeast of Iran reported that washing frequency of once in six months increased from 37.6% to 68.9% following educational intervention. Every six months washing is required to maintain the long-lasting efficacy of LLINs and more frequent washing decreases the efficacy because large proportion of the insecticide is removed during the washing process [32].

According to manufacturer’s instructions bed nets should be dried in shade, However drying in black vinyl bags under direct sunlight is also recommended to improve regeneration of insecticides on the surface of bed nets which is needed for repelling mosquitoes [8, 32].

This study indicated that majority of household dried the Olyset® nets under direct sunlight. Drying the LLINs under direct sunlight is not recommended by manufacturers and should be avoided because it diminishes the biological efficacy of the bed nets.

Based on the findings of this study households in the studied area should be trained on correct procedure of washing and drying of LLINs according to manufacturer’s instructions to protect the potency of LLINs over the time.

## Conclusion

This study highlighted the discrepancy between knowledge about transmission and symptoms of malaria and use of bed nets as a preventive measure. This difference between knowledge and practice is probably due to the fact that although the individual’s knowledge about malaria is high, it has not promoted their attitude towards using the bed nets. Therefore, educational intervention aiming to change the attitude and practice of people is a key element for malarial control in the studied population. In this context mass distribution of LLINs accompanied with education for behaviour change may improve the use of bed nets. Standardized verbal and written education should include information on the regular use and instructions on washing and drying of bed nets, which would improve malaria elimination programme in Iran. Moreover, continuous monitoring and evaluation of LLINs effectiveness is recommended for successful and sustainable malaria elimination programme.

## Electronic supplementary material

Additional file 1: **Survey questionnaire for community knowledge and practices regarding malaria and long-lasting insecticidal nets in an endemic area in Iran.** (DOCX 13 KB)
