# Supplementary material for: Community knowledge and practices regarding malaria and long-lasting insecticidal nets during malaria elimination programme in an endemic area in Iran
Source: Malar J. 2014 Dec 24;13:511. doi: 10.1186/1475-2875-13-511 (PMC4364076; doi:10.1186/1475-2875-13-511)
Supplement: Supplementary file 1 — Additional file 1: Survey questionnaire for community knowledge and practices regarding malaria and long-lasting insecticidal nets in an endemic area in Iran. (DOCX 13 KB) [file 12936_2014_3668_MOESM1_ESM.docx]

Additional file 1 Survey questionnaire for community knowledge and practices regarding malaria and long-lasting insecticidal nets in an endemic area in Iran

**Identification**

**Date: ………..**

**Name of household head……………….**

**Cluster number…………………………**

**Household number……………………...**

**Village name…………………………….**

**NO.**

**Question**

**Categories**

1

Age of interviewee (mother/ daughter/ adult member of household)

………………..years

2

Educational level of interviewee

Illiterate Primary

Secondary High school

University

3

Occupation of interviewee

Housewife

Employed

Self-employed

Farmer/Stockbreeder

Student

4

Family size (Number of persons in household)

………………..

5

Type of house

Cement block house Shed

Tent

6

Do your windows have screen?

Yes No

7

Does your house have water supply?

Yes No

8

Does your house have a water saving container?

Yes No

9

Does your house have electricity power?

Yes No

10

Is your house equipped with an air conditioner?

Yes No

11

Is an animal shelter close to your house?

Yes No

Do you think that malaria is a disease?

Yes No

12

What is the transmission route of malaria? (choosing more than one answer is possible)

Mosquito bites Drinking dirty water

Eating contaminated food

Inhaling polluted air Don’t know

13

What are malaria symptoms?

(choosing more than one answer is possible)

Fever Chill

Fever and chill Bone pain

Abdominal discomfort Don’t know

Have any members of your family been infected by malaria parasites during last 3 years?

Yes No

14

What do you do for malaria prevention?

(choosing more than one answer is possible)

Use of long- lasting insecticidal nets

Use of indoor residual spraying

Chemoprophylaxis

Use of door/window screens

Others Noting

15

Where do Anopheline mosquitoes breed?

(choosing more than one answer is possible)

Stagnant water Rubbish

Don’t know Others

16

What is the source of your information about malaria?

(choosing more than one answer is possible)

Health workers Radio/TV

Religious leader Newspaper/Book

17

18

Are you interested in participating in a malaria control programme as a volunteer?

Yes No

19

Did you receive bet nets from government?

Yes No

20

What are your reason/reasons for use of bet nets?

Prevention of mosquito nuisance

Prevention of other insects nuisance

Prevention of scorpion stings

Others

21

How often do you use bed nets?

*Regularly (Bed net is being used every night)

Irregularly (sometimes bed net use is missed)

Not using

22

Who sleeps under the bed nets?

All family members

Children Father and mother

23

How long do you use bed nets during the night?

All the time at night

Only when sleeping

24

How often do you wash your bed nets?

Once in a month

Once in a six months

Once in a year

Not washing

25

How do you dry the bed nets after washing?

Dry in the sunlight Dry in the shade

***It was checked visually in the morning hours by interviewers**
